# MDMA-Based Psychotherapy in Treatment-Resistant Post-Traumatic Stress Disorder (PTSD): A Brief Narrative Overview of Current Evidence

**DOI:** 10.3390/diseases11040159

**Published:** 2023-11-03

**Authors:** Kainat Riaz, Sejal Suneel, Mohammad Hamza Bin Abdul Malik, Tooba Kashif, Irfan Ullah, Abdul Waris, Marco Di Nicola, Marianna Mazza, Gabriele Sani, Giovanni Martinotti, Domenico De Berardis

**Affiliations:** 1 Dow Medical College, Dow University of Health Sciences, Karachi 75700, Pakistan; kainat.riaz18@dmc.duhs.edu.pk (K.R.); sejalrani175@gmail.com (S.S.); 2 Services Institute of Medical Sciences, Lahore 54000, Pakistan; m.hamzabinmalik@gmail.com; 3 Jinnah Sindh Medical University, Karachi 75510, Pakistan; toobakashif501@gmail.com; 4 Kabir Medical College, Gandhara University, Peshawar 25120, Pakistan; irfanullahecp2@gmail.com (I.U.); dwaris515@gmail.com (A.W.); 5 Department of Geriatrics, Neuroscience and Orthopedics, Fondazione Policlinico Universitario A. Gemelli IRCCS, Università Cattolica del Sacro Cuore, 00168 Rome, Italy; marco.dinicola@policlinicogemelli.it (M.D.N.); marianna.mazza@policlinicogemelli.it (M.M.); gabriele.sani@unicatt.it (G.S.); 6 Department of Neurosciences, Imaging, and Clinical Sciences, University G. D’Annunzio, 66100 Chieti-Pescara, Italy; giovanni.martinotti@gmail.com; 7 Department of Psychiatry, Azienda Sanitaria Locale 4, 64100 Teramo, Italy; 8 School of Nursing, University of L’Aquila, 67100 L’Aquila, Italy; 9 International Centre for Education and Research in Neuropsychiatry, Samara State Medical University, 443100 Samara, Russia

**Keywords:** post-traumatic stress disorder, MDMA, psychotherapy, breakthrough therapy

## Abstract

Post-traumatic stress disorder (PTSD) is a debilitating mental health disorder that causes significant dysfunction in individuals. Currently, there are many approved pharmacotherapy and psychotherapy treatment options for PTSD, but unfortunately, half of the patients do not respond to traditional therapies. In this article, we review clinical trials and research on 3,4-methylenedioxymethamphetamine (MDMA)-assisted psychotherapy in PTSD patients, its pharmacokinetics, and current treatment guidelines for PTSD. Our findings are based on the results of the efficacy of MDMA-assisted psychotherapy from six phase II randomized controlled trials. MDMA-assisted psychotherapy for PTSD has received the “breakthrough therapy” designation from the FDA. MDMA can reduce PTSD symptoms even in treatment-resistant cases by increasing certain neurohormones, i.e., dopamine, serotonin, norepinephrine, and oxytocin. It also modulates activities in the brain regions involved in fear and anxiety. Future research is needed to show whether the advantages outweigh the disadvantages and whether its use can be integrated into available treatment options for PTSD.

## 1. Introduction

Post-traumatic stress disorder (PTSD) is a severe mental health disorder that occurs after experiencing single or repeated extreme traumatic events [1]. PTSD is characterized by a combination of hyperarousal symptoms (hypervigilance, anxiety, and sleep disturbance), disturbing re-experiencing of traumatic experiences (intrusive memories, nightmares, or flashbacks), and avoidance symptoms (emotional numbing and withdrawal). PTSD patients exhibit a significant impact on cognition and emotional processing, leading to a decline in the functions of daily living and interpersonal and social relationships [2].

The current treatment for PTSD is either pharmacological or psychotherapy based on the patient’s preference, and, to date, there are only two approved FDA medications for PTSD treatment [3].

Some patients respond effectively to PTSD treatment and experience a reduction in symptoms; however, according to several studies, 40–60% of patients do not respond to treatment adequately [4,5]. Research for effective treatment has been underway for many years to reinforce exposure-based therapy and various other psychotherapies [2].

One such pharmacological drug, 3,4-methylenedioxymethamphetamine (MDMA), has shown promising results in treatment-resistant PTSD. According to a study by Mithoefer et al. [6], even 3.5 years after undergoing an MDMA-assisted psychotherapy trial, patients showed a long-term durability reduction in PTSD [1]. MDMA-assisted psychotherapy is designated as a “breakthrough therapy of treatment-resistant PTSD” [7].

In this brief narrative review article, we discuss what PTSD is and what the current treatments are, with a particular focus on the most effective psychotherapies. Additionally, after discussing the history of MDMA, its mechanism of action, and pharmacokinetics of MDMA, we evaluate the studies on the efficacy of MDMA-assisted psychotherapy in PTSD.

## 2. Literature Search

We followed the methods of previously published papers [8,9] and searched PUBMED and PsycINFO to identify empirical studies that have applied MDMA to assist psychotherapy in PTSD. We searched for articles published before May 2023 in which abstracts included the terms (i) “post-traumatic or traumatic or PTSD or Post-traumatic stress disorder” or “psychopathology,” combined with one of the following terms: “psychotherapy” or “assisted” or “assisted psychotherapy” or “MDMA” or “3,4-methylenedioxymethamphetamine” or “methylenedioxymethamphetamine” and (ii) “MDMA” or “PTSD” or “psychotherapy.”

## 3. What Is PTSD, and How Can We Treat this Disorder?

There are different forms of potentially traumatic experiences that a person undergoes in the course of a lifetime [10,11]. First, there are “minor traumas” or “t’s,” subjectively disturbing experiences characterized by a perceived danger that is not particularly intense. Events such as humiliation suffered or abrupt interactions with significant persons during childhood can be included in this category [12]. Next to these minor traumas are “T-traumas,” i.e., all those events that lead to death or threaten one’s physical integrity or that of loved ones. Finally, significant events, such as natural disasters, abuse, accidents, etc., belong to this category.

The only diagnosis in the DSM-5 [13] that includes trauma as an etiological factor among the diagnostic criteria is “Disorders due to traumatic and stressful situations.” Some examples of these diseases are reactive attachment disorder, uninhibited social engagement disorder, post-traumatic stress disorder (PTSD), acute stress disorder, adaptation disorders, and other disorders with or without additional criteria.

The following conditions must be met for PTSD to develop in an individual [14].

The individual has been exposed to trauma, such as actual or threatened death, serious injury, or sexual assault (criterion A), either directly or indirectly by experiencing the traumatic event themselves or through hearing about a violent or unintentional traumatic event that occurred to a member of their family or a close friend. Additionally, frequent or excessive exposure to the fundamental components of the traumatic event qualifies as traumatizing, as in the case of first responders gathering human remains or police officers being repeatedly exposed to aspects of child abuse.

After the traumatic occurrence, intrusive symptoms, such as recollections, dreams, and flashbacks that cause a complete loss of awareness of the surroundings, develop (criterion B). In addition, when triggers that represent or resemble the trauma occur, there are significant or protracted psychological discomfort and physical responses.

The next criterion consists of persistent avoidance of the traumatic event’s associated stimuli after the catastrophic event (criterion C). This involves internal and external factors that trigger unpleasant memories, thoughts, or feelings connected to or closely associated with the traumatic event. Examples of internal factors include unpleasant memories, beliefs, or feelings related to or closely associated with the traumatic event. External factors include people, places, conversations, activities, objects, and situations.

After the traumatic occurrence, negative changes in feelings and thoughts connected to the traumatic event take place (criterion D). The individual loses track of specific essential details of the traumatic incident and forms ingrained, exaggerated negative views or expectations about the world, other people, or themselves. It is possible to have distorted and persistent opinions regarding the origins or effects of the traumatic incident, which can lead to blaming oneself or others. The inability to feel pleasant emotions, like happiness, contentment, or love, can also indicate a negative emotional state, as can persistent feelings of fear, terror, rage, guilt, or humiliation. Other symptoms include a sharp decline in interest or participation in worthwhile activities.

After the traumatic experience, significant changes in arousal and reactivity linked to it lead to irritable behavior (criterion E). Anger outbursts are typically expressed through verbal or physical aggression toward people or objects; reckless, self-destructive behavior; hypervigilance; exaggerated alarm responses; concentration issues; and sleep-related symptoms, like trouble falling or staying asleep or restless sleep (with little to no provocation).

The reported alterations last for more than a month (criterion F).

The illness results in clinically substantial distress or reduced functioning in essential domains such as social, occupational, or other (criterion G).

The problem is not caused by another medical illness or the physiological effects of a substance like alcohol or drugs (criterion H).

If the symptomatology has been present for less than three months, the disorder is classified as acute; if it lasts longer than three months, it is chronic; and if, on the other hand, it occurs at least six months after the trauma, it is classified as late onset [15].

Concerning the lifetime prevalence of the disorder among the general population, epidemiological studies have estimated it to be between 1% and 10% for women and 5% for men, with variability attributable to the methods of ascertaining and sampling the population used [16]. People with PTSD often fulfill the criteria for at least one other diagnosis in comorbidity [17]. The most common appears to be major depression, with frequency rates of around 46%, followed by other anxiety disorders, particularly panic disorder and social phobia, which affect between 20 and 30% of subjects [18]. A diagnosis of substance abuse or dependence is also prevalent for 52% of men and 28% of women with chronic PTSD [19]. Somatic symptoms that meet the criteria for a diagnosis of somatization disorder may also be present [18]. Another widespread condition, although not an actual diagnosis, is the frequent feeling of guilt experienced by individuals with PTSD concerning their behaviors to ensure their survival, including the fact that they survived when others died, and their reactions after the traumatic event [20,21].

PTSD is a rather pervasive disorder; the sufferer is absorbed in painful memories of the traumatic event or avoids reliving it [22]. He/she has difficulty expressing and experiencing emotions, decreased sexual desire, and a loss of interest in previously pleasurable activities, so much so that he/she gives the impression of thinking only of him/herself [23]. He/she often feels tired and constantly threatened, has sudden outbursts of anger, and is continuously nervous. In addition, he/she has feelings of shame, despair, and guilt. This often impacts interpersonal and work relationships, hindering or making it impossible to lead an everyday life [12]. Social withdrawal and frequent marital conflicts are often observed, leading to relationship breakdown and divorce, as significant others and the partner in this condition can feel neglected, rejected, or unloved [24]. Similarly, the overall symptom picture at work often leads to frequent arguments with colleagues and superiors and a decline in performance, leading to dismissal [24].

Studies on pharmacotherapy alone in treating PTSD have shown a reduced effect compared to integrated psychotherapeutic interventions [25]. However, serotonin reuptake inhibitors, such as fluvoxamine, sertraline, or paroxetine; or serotonin and norepinephrine reuptake inhibitors, such as venlafaxine, are effective in reducing post-traumatic symptoms and are considered to be the first-choice pharmacological treatments [26]. However, antidepressants are not always effective in counteracting insomnia and nightmares; it would be appropriate to use sleep-inducing drugs or atypical antipsychotics for such symptoms [27]. Prazosin effectively reduces nightmares, insomnia, and hyperarousal [28]. Since comorbidity between PTSD and substance abuse, such as alcohol and drugs, is proven to be frequent, it has been seen that the use of an opioid receptor antagonist, such as naltrexone, combined with exposure, significantly reduces both symptoms of PTSD and substance abuse [29]. Thus, it was seen that, in general, all pharmacological categories are used to achieve different effects. Antidepressants can be helpful, but their efficacy seems lower than when used for treating mood disorders [30,31,32,33]. SSRIs seem more effective, whereas mood stabilizers can reduce irritability, impulse control, and flashbacks [34].

Medications must be considered more as an opportunity to nurture patient compliance and not as a treatment in itself; only very rarely do drugs bring about a complete remission of symptoms, so it is essential to combine psychotherapy [35]. Some medications, such as fluoxetine, paroxetine, or venlafaxine, seem to have a positive effect on the symptoms of PTSD. Still, most others do not seem to have significant efficacy, highlighting the need for more research [36]. It has been shown that the remission of symptoms achieved with psychotherapy (particularly Eye Movement Desensitization and Reprocessing, EMDR) remains over the long term [37]. In contrast, using medication alone and its subsequent discontinuation can lead to rapid relapses. However, other studies have pointed out that there is no robust evidence to prove a better efficacy of the combination of psychotherapy and pharmacotherapy than either treatment modality taken alone [26].

A new frontier in treating PTSD is using substances that can enhance the psychotherapy experience and enactment. Among these, exciting data have emerged on the use of MDMA [38].

## 4. Current Psychotherapies in the Treatment of PTSD: Benefits and Limitations

One of the most widely recognized and evidence-based therapies for PTSD is cognitive–behavioral therapy (CBT) [39], and several studies have proven the effectiveness of CBT in reducing PTSD symptoms and improving overall functioning [40,41].

EMDR therapy is another highly effective approach for treating PTSD [42]. EMDR integrates elements of CBT with bilateral stimulation, typically achieved by asking patients to focus on a therapist’s moving hand or using alternating sounds [43]. This bilateral stimulation is theorized to help reprocess traumatic memories, thereby reducing the distress associated with them [44]. EMDR also incorporates cognitive restructuring techniques, fostering a more positive belief system surrounding the traumatic event [45]. Research has shown that EMDR significantly reduces PTSD symptoms and helps individuals experience significant improvement in their overall well-being [42,46].

Narrative exposure therapy (NET) is a psychotherapeutic approach that is designed specifically for individuals who have experienced complex and multiple traumas [47]. It emphasizes the importance of reconstructing a coherent narrative around the traumatic events and integrating it into the individual’s life story [48]. NET combines exposure therapy, cognitive restructuring, and cognitive processing therapy [49]. By creating a detailed and organized account of the traumatic experiences, NET allows individuals to gain a renewed sense of control and mastery over their memories and emotions [50]. Multiple studies have demonstrated the efficacy of NET in reducing PTSD symptoms and improving overall psychological functioning [51,52].

Alongside these therapies, acceptance and commitment therapy (ACT) has shown promising results in treating PTSD [53]. ACT focuses on helping individuals accept their traumatic experiences and associated emotional responses without trying to suppress or change them. Through mindfulness techniques and identifying personal values, ACT helps individuals develop greater dynamic flexibility and engage in behaviors that align with their core values. This approach enables individuals to focus on living a meaningful life despite their traumatic past. Recent studies have indicated that ACT can help reduce PTSD symptoms, improve well-being, and enhance overall psychological flexibility [53,54].

Several arts-based psychotherapies help treat PTSD, especially at the initial stage and in addition to pharmacological treatment [55]. As PTSD patients are often overwhelmed by the traumatic events they experienced, visual art or poetry can help them to imagine, make sense of, and explain the overwhelming thoughts and sentiments in more contained ways [56]. Using visual arts and poetry in psychotherapy can ease mental processes, which can benefit the treatment (i.e., releasing and recognizing one’s emotions and thoughts, facing one’s fears, and establishing a new sense of oneself) [57]. Poetry and visual arts can be used for various treatment intentions, which benefit a PTSD patient. The patients can gain inspiration; express thoughts and feelings; construct new life stories; play and engage in creative experiences; and engage in meditation and prayer [58].

Alternative therapies such as art therapy, writing therapy, dance/movement therapy, and drama therapy can further help patients with PTSD—especially treatment-resistant subjects—to improve their negative beliefs, reduce hyperarousal, focus on the positive things in life, and create alternative narratives and engage in creative coping [59]. It is recognized that pleasure and enjoyment are important preferences for arts therapies, and it has been suggested that people making non-consequential decisions will do so either on the basis of mental pleasure or to minimize mental displeasure [60]. In the arts therapies, pleasure and playfulness are more important than in other forms of therapy, as there is an emphasis on using creativity to explore different cognitive or emotional experiences [59,61].

While psychotherapies can be highly effective in treating PTSD, it is essential to acknowledge that they do not work for everyone and that treatment outcomes can vary. There are several reasons why psychotherapies can fail or be less successful in treating PTSD [62]. One primary reason for treatment failure is the complexity and severity of the traumatic experience. Trauma can manifest in different ways and vary significantly in its impact on individuals. Someone may have experienced multiple traumas or have long-standing traumatic experiences that make it more challenging to address and treat their PTSD symptoms effectively [63].

Comorbid mental health conditions can also contribute to treatment failure [64]. PTSD often co-occurs with mental health disorders such as depression, anxiety, or substance abuse, and these comorbid conditions can complicate treatment and require concurrent or additional therapies to address all aspects of the individual’s mental health effectively [16]. The lack of engagement and motivation in therapy can also lead to treatment failure. For therapy to be effective, individuals must actively participate and commit to the treatment process: if individuals are resistant or disengaged, it can impede progress and hinder the therapeutic relationship [62].

In some cases, limited access to appropriate care can hinder the success of psychotherapies [65]. Accessibility issues, such as financial constraints, limited availability of qualified providers, or geographic location, may limit an individual’s ability to access and receive consistent and effective psychotherapy.

Individual differences can also influence the failure of psychotherapies for PTSD [62]. Some individuals have personal characteristics, coping styles, or cognitive patterns that make them less responsive to specific therapeutic approaches. The therapeutic relationship and rapport between the therapist and client can also impact treatment outcomes [66]. Despite these challenges, it is essential to note that alternative options are often available even when psychotherapies fail or are less successful. These can include trying different therapeutic approaches; combining therapies; or exploring other treatment modalities, such as medication or neuromodulation techniques. Mental health professionals must be flexible, responsive, and adaptable, tailoring the treatment to each individual’s unique needs and circumstances.

An “alternative” approach using MDMA during psychotherapy, if available, is an excellent option to treat resistant cases and overcome barriers [67].

## 5. History of MDMA, Chemistry, and Pharmacokinetics

MDMA was synthesized in 1912 by the German company Merck when researchers were trying to develop a vasoconstrictor to stop bleeding [68,69]. However, they accidentally discovered MDMA instead, called methylsafrylaminc [70]. Between 1970 and 1980, MDMA was used by some psychiatrists, as they believed that it resulted in effective communication with patients, even though it was not approved by the FDA for human use or formal clinical trials [71,72].

In 1985, the Drug Enforcement Agency (DEA) observed MDMA abuse as a nationwide problem and announced an emergency ban on MDMA, which was classified as a “Schedule I drug” [71]. Then, in the mid-1990s, the US FDA approved the phase I trial of MDMA in healthy volunteers for the first time [4].

Currently, the use of MDMA is restricted because of its high risk of abuse potential and side effects, and clinicians should weigh the risks vs. benefits of such a treatment. Although MDMA-assisted psychotherapy can be considered in reactive disorders such as PTSD, it can exacerbate some other mental health disorders, so it should be used cautiously [73].

MDMA is a ring-substituted amphetamine that is structurally similar to methamphetamine and mescaline. It is usually present in two optical isomers, with the dextrorotatory form, S (+) MDMA, being more potent in the central nervous system (CNS) [74]. The psychostimulant and empathic effects of MDMA are caused by the S (+) isomer, whereas the R isomer is responsible for its hallucinogenic properties [75]. It is commonly given via the oral route, but it is also inhaled for a faster effect. MDMA is rapidly absorbed in the bloodstream, and two metabolic pathways metabolize it. The central metabolism pathway starts with demethylation by cytochrome P450 (CYP) 2D6 to intermediate 3,4-dihydroxy methamphetamine (HHMA). Then, after conversion to 4-hydroxy-3-methoxyamphetamine (HMA) following many steps in a minor pathway, it is N-demethylated to 3,4-methylenedioxyamphetamine MDA (which is sometimes used recreationally) before ending up in HMA [76]. The combination of MDMA and HHMA is accountable for 58% of the total drug in the urine [76,77].

When taken orally, its effects begin in 30 to 60 min and last up to 8 h, and its peak plasma concentrations occur 2 to 4 h after an oral dose. The half-life of MDMA is between 7.7 and 8.6 h. The 11 S (+) isomer has a 30% shorter half-life than the levorotatory form because its metabolism is faster and more extensive [78,79].

The evidence obtained in experimental animal models concerning the action of MDMA shows the extent to which exposure to ecstasy can induce alterations in the function of the serotonergic system, and these alterations are of a persistent nature, i.e., not immediately reversible upon discontinuation of the drug [80]. It has been shown that chronic exposure to ecstasy is capable of producing presynaptic alterations on serotonergic axon terminals, with an action involving ATP synthesis and the Na-K pump at the synaptic level [81]. MDMA causes an inhibition of the serotonin transporter, along with a blockade of brain monoamine reuptake and the possible risk of a “depletion” of serotonin stores [82]. This can result in a so-called “always open door” condition for serotonin and an inability to store it in presynaptic vesicles [83]. A consistent study has shown serotonin deficiency and its metabolites in experimental animals. According to some studies, MDMA produces persistent serotonin depletion in the rat brain, particularly in the basal nuclei and striatum [84]. Others have found reduced serotonin and 5-hydroxy-indoleacetic acid levels in mice exposed to ecstasy in the frontal cortex, hippocampus, and striatum [85]. An actual degeneration of the distal elements of the axon has been hypothesized in connection with the documented reduction of various markers of the serotonergic axon, including serotonin, 5-hydroxy-indoleacetic acid, the enzyme tryptophan hydroxylase, and the transporter for serotonin [86]. The term “denervation” has been suggested to define the substantial damage that ecstasy produces on serotonergic neurons [75].

Despite this, however, not all evil leads to harm. Paracelsus is considered the father of modern medicinal chemistry and toxicology. It was Paracelsus who said, “It is the dose that makes the poison” (in Latin, “Sola dosis facit venenum”). Even today, Paracelsus’s quote is true, especially for several specific substances, such as ketamine, psilocybin, Lysergic Acid Diethylamide (LSD), and MDMA. These substances, under strict control, at the proper doses, and in medical settings are an essential breakthrough in treating complex disorders such as major depression, substance abuse, and, obviously, PTSD. Therefore, MDMA, when used as a medication and not as a drug of abuse, can be helpful in treating several psychiatric disorders.

## 6. MDMA-Assisted Psychotherapy in PTSD: An Overview

Trauma-focused cognitive–behavioral therapy (CBT) and eye movement desensitization and reprocessing (EMDR) are the most common treatments for PTSD. A meta-analysis of published clinical trials from 1980 to 2003 on the clinical efficacy of psychotherapy for PTSD treatment by Bradley et al. showed that around 67% of patients completed treatment, and the recovery rate for PTSD averaged 50–60% who received therapy [87]. PTSD is a complicated disorder that needs psychological and pharmacological intervention. Currently, only two similar-acting medication therapies—Sertraline and Fluoxetine—have been approved by the FDA [27,88].

All current MDMA-based psychotherapies are randomized controlled trials that are monitored by the FDA and overseen by the Institutional Review Board (IRB) [73,89]. Due to the schedule of MDMA, a level-controlled substance review committee is required. MDMA-assisted psychotherapy utilizes single-dose MDMA administration once a month, on two or three occasions, followed by preparatory and psychotherapy sessions [73,90]. MDMA is a psychoactive compound that is commonly misused under the name of the street drug “Ecstasy,” which reduces the uptake of amines such as serotonin, dopamine, and norepinephrine from presynaptic terminals, thus contributing to overcoming anxiety and increased bonding [91,92].

A clinical phase III trial conducted by Ot’alora et al. [93] also reported that MDMA-assisted psychotherapy is an efficacious treatment for PTSD. The randomized double-blind trial of 28 people with chronic PTSD also included the comparison of two active doses, i.e., 125 mg and 100 mg, with a low amount of 40 mg administered during 8-h psychotherapy sessions. Changes in the clinically administered PTSD scores one month after the two sessions of MDMA served as a primary outcome, and yearly follow-up assessments also occurred after the final dose. The results showed that the active group cohort significantly reduced the clinically administered PTSD scores with a mean score change of −26.3 with 125 mg, −24.4 with 100 mg, and −11.5 with 40 mg. However, statistical significance was reached only in the per-protocol set (*p* = 0.03). PTSD symptoms were well-controlled at the yearly follow-up assessment (*p* < 0.001), with 76% (*n* = 25/28) not meeting post-traumatic stress disorder criteria, and no side effects from the MDMA were observed.

Administration of MDMA in treatment-resistant PTSD from a psychotherapeutic perspective, its efficacy is hypothesized to result from its positive psychological effects. The positive outcomes of MDMA in treatment-resistant PTSD therapy show a strong relationship with the strength of the therapeutic alliance [94]. A 2014 study by Doukas et al. [95] shows that forming trust-based relationships is often tricky for PTSD patients. Many PTSD patients experience a short period of optimal excitation, often leading to distress and sometimes dissociation [96]. Many of these challenges are alleviated by MDMA, as MDMA exerts its effects by increasing neurohormones, e.g., oxytocin; prolactin; cortisol; and monoamine neurotransmitter serotonin [97]. Oxytocin is suggested to play a role in accurately perceiving emotion, affiliation, and trust [98,99], proving beneficial in PTSD patients. It helps patients recall traumatic experiences in emotional engagement [100,101]. Elevated oxytocin levels improve social bonding, increase trust, and decrease amygdala activation in patients with PTSD [102,103]. Threatening interpretations are raised by negative emotional states. MDMA causes increased serotonin levels, which diminish the negative emotional state by enhancing self-confidence and reducing feelings of anxiety and depression. Hence, this helps PTSD patients to approach past experiences differently with ease. MDMA helps in the fear-reduction process, according to the Emotional Processing Theory (EPT) of exposure therapy [96,104].

Investigations were conducted on MDMA-assisted psychotherapy for PTSD patients who had failed previous treatments. Marseille et al. [72] conducted a cross-sectional study with 29 participants who received forgiveness treatment and 29 who received an inactive placebo as part of the control group. The results from the pre- and post-tests suggested that MDMA-assisted psychotherapy is helpful for those who have gone through psychological trauma but cannot find relief from their difficulties by using conventional treatments.

MDMA-assisted psychotherapy’s cost-effectiveness for treating chronic, treatment-resistant PTSD was examined [105]. A decision-analytic Markov model was created as part of an experimental research design to demonstrate the costs and health benefits of employing MDMA-assisted psychotherapy to treat patients with chronic, severe, or extreme treatment-resistant PTSD. In six double-blind phase 2 trials, MDMA-assisted psychotherapy involved an average of 2.5 trauma-focused psychotherapy sessions lasting 90 min, followed by two sessions, each lasting eight hours with MDMA (mean dose: 125 mg), and then an average of 3.5 integration sessions. The control group received either an inert placebo or 25–40 mg of MDMA. The results demonstrated that MDMA-assisted psychotherapy appears to be financially advantageous [105]. Mitchell et al. [106] randomized 90 PTSD patients (most with common comorbidities, such as dissociation, depression, alcohol and substance-use disorders, and juvenile trauma) to receive manualized therapy with MDMA or with a placebo, combined with three preparatory and nine integrative therapy sessions. They found that MDMA-assisted treatment was effective and well-tolerated in people with severe PTSD, even including those with comorbidities. Moreover, MDMA-assisted psychotherapy for severe PTSD also improves alcohol use without increasing the risk of illicit drug use [107].

Interestingly, a study employed an Interpretative Phenomenological Analysis (IPA) for seven participants who met the criteria for severe PTSD to develop a better understanding of MDMA-assisted therapy and its efficacy [108]. The subjects reported significantly improved conflict tolerance, connectedness, and positive emotions. In addition, they showed increased acceptance, self-forgiveness, and self-empathy, which are vital in addressing moral injury and the feelings of guilt and shame common in severe PTSD.

The abovementioned study also investigated how the use of psychedelics eased the effects of racial PTSD among black, indigenous, and other people of color (BIPOC) after experiencing racism [109]. Questions on encounters with racism, mental health symptoms, and immediate and long-lasting psychedelic effects were included in the cross-sectional online study survey. In addition, retrospective reports of symptoms from the 30 days before and 30 days after an experience with psilocybin, lysergic acid diethylamide, or MDMA were used to measure changes in mental health. Three hundred thirteen volunteers from various BIPOC populations in the US and Canada were recruited for the study. Compared to before and after the psychedelic experience, the findings showed a substantial and modest reduction in PTSD symptoms.

## 7. Discussion

MDMA-assisted psychotherapy has emerged as a promising and innovative treatment approach for individuals with PTSD, and MDMA, when used in a therapeutic context, is always administered in controlled settings under the guidance of trained therapists and not used recreationally or without psychological support [110]. The substance enhances the therapeutic process by inducing increased empathy and relaxation and reducing the fear response, thus helping individuals with PTSD to confront and process traumatic memories and emotions in a safe and supportive environment [111].

As described above, several studies have shown positive results and, for example, the Multidisciplinary Association for Psychedelic Studies (MAPS) conducted by Mitchell et al. [106] (see above) demonstrated significant reductions in PTSD symptom severity after MDMA-assisted psychotherapy, with some participants reporting long-lasting benefits even years after treatment, thus indicating the potential of MDMA to catalyze profound and enduring healing in individuals struggling with the debilitating effects of trauma. MDMA-assisted psychotherapy allows individuals to access and work through deeply buried emotions and memories that are often difficult to explore without overwhelming distress [38]. The heightened emotional openness and trust the substance fosters can enhance the therapeutic alliance and facilitate breakthroughs in understanding and healing [112]. The treatment involves a series of therapy sessions, including preparatory and integration sessions, to ensure the consolidation and application of insights gained during MDMA-assisted sessions [108]. In addition, beyond reducing symptoms, MDMA-assisted psychotherapy has been shown to enhance therapeutic processes [113]. The empathogenic and entactogenic effects of MDMA help individuals with PTSD develop a sense of safety and trust within the therapeutic setting, facilitating the exploration and processing of traumatic memories and emotions that are often at the core of their condition [111]. MDMA is thought to increase feelings of connection, empathy, and self-compassion, which can contribute to healing and integration.

The MDMA-assisted therapy follows the principles of the client-centered therapy and is determined by the therapeutic relationship established between the therapist and patient [114]. According to this approach, the psychotherapist does not possess protocol intervention techniques and therefore is free to interact with the client’s individuality through the mediation of the effects of MDMA [115]. We believe that the relationship in MDMA-assisted psychotherapy must follow a certain pattern characterized by the following [116]:

- Non-directedness: The relationship that is established between therapist and client is an equal one; the therapist incites the client to use his or her personal resources to find a solution to the problem presented, using MDMA as a facilitator [117].
- Empathy: For the relationship to bring results, it is necessary for the therapist to put on the client’s shoes and attempt to see the world through the client’s eyes, abandoning his or her own personal patterns.
- Acceptance: The therapist accepts the client’s thoughts and behaviors unconditionally and therefore listens actively and without enacting prejudice.

MDMA-client-centered therapy is particularly suitable in cases where people are unable to get in touch with their experiences and recognize their emotions, and this, especially in treatment-resistant PTSD, results in a kind of inner conflict and inauthenticity, which leads the person to not fully be himself/herself in the relationship [118]. Carl Rogers has called this state “incongruence,” which does not allow the individual to grow positively or make his or her own choices optimally [119]. A strong therapeutic alliance is needed in MDMA-assisted therapy in order to achieve a good client-centered therapy that aims at encouraging the patient to open up freely to the therapist in an authentic way [120]. Moreover, through such a therapeutic process mediated by MDMA, it is possible to empathically understand how the other constructs his or her relationship with the self, others, and the world [115].

While the results are promising, cautious considerations must be made regarding safety and ethics [121]. MDMA has potential risks and side effects, including cardiovascular and neurotoxicity, which must be carefully monitored and managed. Additionally, ethical considerations must guide the responsible use of MDMA, including informed consent, the exclusion of individuals with certain medical conditions, and the importance of trained and honest therapists overseeing the treatment [38].

Despite some concerns, MDMA-assisted psychotherapy represents a groundbreaking approach to treating PTSD. When used in a controlled therapeutic context, the substance facilitates emotional breakthroughs, enhances the therapeutic process, and reduces PTSD symptoms [110]. While further research and integration into mainstream clinical practice are needed, MDMA-assisted psychotherapy holds immense promise for individuals seeking relief from the devastating effects of PTSD [122]. This innovative treatment approach opens doors for healing and transformation, providing hope and a renewed quality of life for those who have previously struggled to find effective treatments for their PTSD [73].

The current narrative review has limitations. It reviewed the literature about MDMA-assisted psychotherapy narratively to summarize recent findings for a broad audience (not only psychiatrists), but a systematic review and meta-analysis on this topic would have provided better and focused insights into this.

## 8. Conclusions

The MDMA-assisted psychotherapy trial has had long-term durability for the reduction of symptoms in PTSD. MDMA-assisted psychotherapy is designated as a breakthrough therapy of treatment-resistant PTSD. The therapeutic alliance between the patients and the therapists is essential to establishing a holistic client-centered therapy that is the key to obtaining the desired effects of MDMA-assisted psychotherapy for both patients and clinicians.

However, the use of MDMA is now restricted because of its high risk of abuse potential and side effects. Therefore, the clinician should always weigh the risks vs. benefits of treatment. However, MDMA-assisted psychotherapy, if available, must be considered for treatment-resistant PTSD, but it can even exacerbate some other mental health disorders, so it should be used cautiously. Currently, MDMA-based psychotherapies are randomized controlled trials monitored by the FDA and overseen by the IRB.
